# Systematic Review of Left Ventricular Remodeling in Response to Hypoglycemic Medications: Assessing Changes in End-Systolic and End-Diastolic Diameters

**DOI:** 10.3390/biomedicines12081791

**Published:** 2024-08-07

**Authors:** Bogdan-Flaviu Buz, Rodica Anamaria Negrean, Florina Caruntu, Tudor Parvanescu, Milena Slovenski, Mirela Cleopatra Tomescu, Diana-Aurora Arnautu

**Affiliations:** 1 Doctoral School, Victor Babes University of Medicine and Pharmacy, 300041 Timisoara, Romania; flaviu-bogdan.buz@umft.ro; 2 Multidisciplinary Heart Research Center, Victor Babes University of Medicine and Pharmacy, 300041 Timisoara, Romania; caruntu.florina@umft.ro (F.C.); parvanescu.tudor@umft.ro (T.P.); milena.slovenski@umft.ro (M.S.); tomescu.mirela@umft.ro (M.C.T.); aurora.bordejevic@umft.ro (D.-A.A.); 3 Department of Internal Medicine, Victor Babes University of Medicine and Pharmacy, 300041 Timisoara, Romania; 4 Cardiology Clinic, Institute of Cardiovascular Diseases, 300310 Timisoara, Romania; 5 Department of Preclinical Disciplines, Faculty of Medicine and Pharmacy, University of Oradea, 410073 Oradea, Romania; 6 Cardiology Clinic, Clinical Municipal Emergency Hospital, 300040 Timisoara, Romania

**Keywords:** cardiology, systematic review, heart failure

## Abstract

Hypoglycemic medications are widely used in managing diabetes mellitus, with emerging evidence suggesting their role in cardiac reverse remodeling. This systematic review aims to quantitatively synthesize data regarding the impact of these medications on left ventricular end-diastolic diameter (LVEDD) and end-systolic diameter (LVESD), and to evaluate the clinical relevance of these changes in promoting favorable cardiac outcomes. We conducted a comprehensive search across PubMed, Scopus, and the Web of Science up to 22 April 2024, selecting studies based on inclusion criteria that focused on the impact of hypoglycemic medications on LVEDD and LVESD in patients with diabetes. Studies were selected through a rigorous process, adhering to PRISMA guidelines, and involving various designs including randomized controlled trials and observational studies. The main outcomes were changes in LVEDD and LVESD measured by validated cardiac imaging techniques. A total of ten studies met the inclusion criteria, involving a total of 1180 patients. Treatment durations ranged from 3 to 24 months. Significant improvements in cardiac dimensions were noted with some medications. For instance, Liraglutide treatment over three months significantly improved LVEF from 47.2% to 57.2% and reduced LVEDD and LVESD from 46.5 mm to 45.2 mm and 35.2 mm to 32.7 mm, respectively. In contrast, other medications like Sitagliptin showed minimal impact over 24 months. On average, hypoglycemic medications reduced LVEDD from 58.2 mm to 55.0 mm and LVESD from 48.3 mm to 44.3 mm, with a mean improvement in LVEF from 38.9% to 43.8%. Hypoglycemic medications contribute variably to cardiac reverse remodeling. Medications such as Liraglutide and Dapagliflozin demonstrate significant potential in improving cardiac dimensions and function, indicating their utility beyond glycemic control. This review highlights the need for tailored treatment approaches to maximize cardiac outcomes in patients with diabetes, suggesting a broader therapeutic role for these agents.

## 1. Introduction

Cardiac reverse remodeling refers to structural and functional improvements in the heart, particularly the left ventricle, observed after targeted medical interventions in conditions like heart failure [1,2,3]. With heart failure affecting approximately 26 million people globally, and its prevalence increasing with an aging population, the need for effective treatments is critical [4,5]. Measures of left ventricular end-systolic (LVESD) and end-diastolic diameters (LVEDD) are essential in monitoring these improvements, directly correlating with patient outcomes [6,7].

Hypoglycemic medications, notably those within the SGLT2 inhibitor class, extend beyond glycemic control to potentially influence cardiac outcomes [8,9]. Research suggests these medications might facilitate cardiac structural improvements through mechanisms like reduced cardiac load and enhanced myocardial metabolism [10,11]. Given the convergence of diabetes and heart failure, where approximately 30% of heart failure patients also suffer from diabetes, these findings are particularly significant [12,13].

Speckle tracking echocardiography (STE) is a prominent example, offering a sophisticated approach to evaluate myocardial deformation and function without relying on geometric assumptions about the heart’s shape [14]. This technique tracks the natural acoustic markers or ‘speckles’ within the myocardial tissue throughout the cardiac cycle, allowing for the detailed analysis of left ventricular strain patterns [15]. Furthermore, the advent of three-dimensional echocardiography and cardiac magnetic resonance imaging (MRI) provides even more detailed and accurate assessments of ventricular volumes and function [16].

Based on these measurement methods, recent studies have shown varying degrees of left ventricular remodeling in patients using hypoglycemic medications [17]. For instance, clinical trials involving SGLT2 inhibitors have reported reductions in left ventricular indices, suggesting potential reverse remodeling effects [18]. In the assessment of left ventricular dimensions such as LVESD and LVEDD, advanced imaging techniques have significantly enhanced the precision and reliability of measurements. Therefore, the hypothesis of this systematic review is that hypoglycemic medications contribute to significant cardiac reverse remodeling in patients with diabetes, as evidenced by reductions in LVESD and LVEDD. The objectives were to quantitatively synthesize data on the impact of these medications on left ventricular diameters and to evaluate the clinical relevance of these changes, aiming to pinpoint the most effective agents promoting favorable cardiac outcomes.

## 2. Materials and Methods

### 2.1. Eligibility Criteria

This systematic review selected studies for inclusion based on the following criteria: (1) patients diagnosed with diabetes mellitus and treated with hypoglycemic medications, assessing their effects on LVESD and LVEDD; (2) research specifically investigating the cardiac reverse remodeling effects associated with the use of these medications, with a particular focus on changes in LVESD and LVEDD as primary outcomes; (3) a comprehensive range of study designs, including randomized controlled trials, observational studies, cohort studies, case-control studies, and cross-sectional studies; (4) studies employing validated cardiac imaging techniques, such as echocardiography or magnetic resonance imaging, to measure changes in LV dimensions accurately; and (5) only peer-reviewed articles published in English.

The exclusion criteria were the following: (1) studies not involving human participants, such as in vitro or animal studies, except where preclinical data were used to support clinical findings; (2) research not specifically examining the impact of hypoglycemic medications on cardiac dimensions or studies that did not differentiate the effects of these medications from other therapeutic interventions; (3) studies that failed to provide clear, quantifiable outcomes related to changes in LVESD and LVEDD or lacked sufficient detail for a comprehensive assessment; (4) gray literature, including non-peer-reviewed articles, preprints, conference abstracts, general reviews, commentaries, and editorials, to ensure consistency and reliability of the analyzed data; and (5) studies deemed low-quality based on predetermined, quantifiable metrics.

In this study, we prioritized cardiac remodeling, specifically left ventricular dimension changes, as the primary outcome over direct outcomes like mortality and hospitalization. This decision is grounded in established correlations between ventricular remodeling indices and clinical endpoints in heart failure. Reductions in left ventricular end-systolic and end-diastolic diameters are robust predictors of improved survival and reduced morbidity. These parameters not only offer insights into the direct effects of therapeutic interventions on cardiac morphology but also serve as prognostic indicators, allowing for the early assessment of treatment efficacy. Therefore, focusing on cardiac remodeling provides crucial, actionable data on the cardioprotective effects of hypoglycemic medications in patients with coexisting diabetes and heart failure.

### 2.2. Information Sources

The primary information sources for this systematic review were the electronic databases PubMed, Scopus, and the Web of Science. The literature search specifically targeted publications up to the initial search date, 22 April 2024. The search was designed to encompass a broad spectrum of studies, including those that evaluate clinical outcomes, patient demographics, treatment modalities, and specifically the impact of hypoglycemic medications on cardiac reverse remodeling, focusing on changes in LVESD and LVEDD.

### 2.3. Search Strategy

The search strategy for this systematic review was meticulously developed using an array of keywords and phrases that are closely aligned with the objectives of the study, which centers on the influence of hypoglycemic medications on cardiac reverse remodeling, particularly the changes in left ventricular end-systolic diameter and end-diastolic diameter. Key search terms included “diabetes mellitus”, “type 2 diabetes”, “hypoglycemic medications”, “antidiabetic drugs”, “cardiac reverse remodeling”, “cardiac structure changes”, “left ventricular dimensions”, “LVESD”, “LVEDD”, “ left ventricular end-systolic diameter”, “ left ventricular end-diastolic diameter”, “cardiac function”, “heart structure modification”, “speckle tracking echocardiography”, “3D echocardiography”, “cardiac MRI”, “clinical outcomes”, “SGLT2 inhibitors”, “GLP-1 receptor agonists”, “DPP-4 inhibitors”, and “metabolic effects on heart.”

These terms were integrated using Boolean operators (AND, OR, NOT) to construct a comprehensive search string. The search included relevant Medical Subject Headings (MeSH) to ensure the inclusion of all the pertinent literature. The search string was thus formulated: (((“diabetes mellitus” OR “type 2 diabetes”) AND (“hypoglycemic medications” OR “SGLT2 inhibitors” OR “GLP-1 receptor agonists” OR “DPP-4 inhibitors”) AND (“cardiac reverse remodeling” OR “cardiac function” OR “heart structure modification”) AND (“left ventricular dimensions” OR “LVESD” OR “LVEDD”) AND (“echocardiography” OR “speckle tracking” OR “3D echocardiography” OR “cardiac MRI”) AND (“clinical outcomes” OR “heart failure” OR “cardiovascular effects” OR “metabolic effects”))).

### 2.4. Selection Process

Consistent with the Preferred Reporting Items for Systematic Reviews and Meta-Analyses (PRISMA) guidelines [19], the selection process for this review was designed to ensure both transparency and methodological rigor. Initially, all retrieved articles were independently screened by two reviewers to confirm their compliance with the pre-established inclusion and exclusion criteria. Discrepancies between reviewers during this preliminary phase were resolved through a consensus discussion or, when necessary, the intervention of a third reviewer. To enhance the credibility and reproducibility of our review process, the entire protocol, including detailed methodologies, was registered and is available on the Open Science Framework (OSF) under the registration code osf.io/tebvq.

### 2.5. Data Collection Process

The data collection phase began with the removal of duplicate records from the initially retrieved articles. Following this, two independent reviewers undertook a meticulous screening of abstracts and, where required, full texts to determine the eligibility of each study according to the set inclusion and exclusion criteria. During this stage, any disagreements between the reviewers were thoroughly discussed until a consensus was reached. For unresolved discrepancies, a third reviewer was brought in to make the final decision. This structured approach ensured a comprehensive and unbiased selection of studies for inclusion in the systematic review.

### 2.6. Data Items

For this systematic review, primary data items collected included cardiac measurements such as left ventricular ejection fraction (LVEF), LVEDD, and LVESD. These metrics were used to assess the impact of hypoglycemic medications on cardiac reverse remodeling. Secondary data encompassed study characteristics such as design and quality, geographic location, patient demographics (age, gender, and comorbidities), and specifics of hypoglycemic treatment (medication type and duration). Treatment specifics, including the type of hypoglycemic medication used (GLP-1 receptor agonists, DPP-4 inhibitors, and SGLT-2 inhibitors), and the treatment duration were recorded to evaluate the differential effects of various drug classes on cardiac outcomes. Outcomes were further interpreted in terms of improvement or deterioration in cardiac function, with a specific focus on statistically significant changes observed over the treatment period.

### 2.7. Risk of Bias and Quality Assessment

The assessment of the methodological quality of observational studies was conducted employing the Newcastle–Ottawa Scale [20]. This scale scrutinizes three fundamental aspects: the selection criteria for the study groups, their comparability, and the determination of exposure or outcome relevant to case-control or cohort studies, respectively. Studies receive stars for performance in these categories, accumulating a total score that categorizes the quality of each study as low, medium, or high.

## 3. Results

### 3.1. Study Selection and Study Characteristics

**Figure 1:**
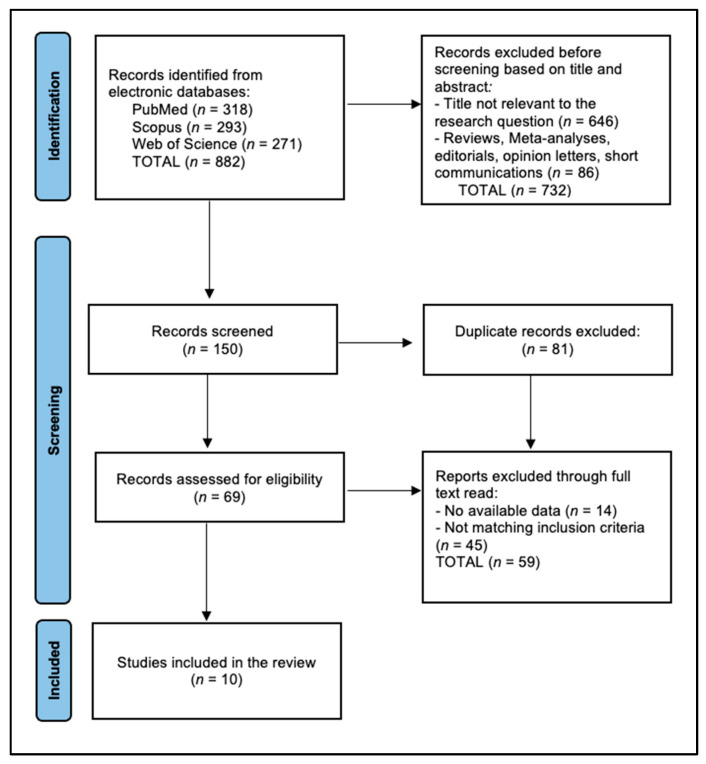
PRISMA flow diagram.

Following an initial literature search, 882 articles were identified. Preliminary screening based on titles and abstracts led to the exclusion of 732 entries deemed irrelevant. A subsequent in-depth examination of the remaining articles resulted in the removal of 81 duplicates and 59 articles that either did not meet the inclusion criteria or lacked essential data. Ultimately, the systematic review included ten studies, conducted between 2015 and 2024, which are systematically presented in Figure 1.

Table 1 outlines the characteristics of the ten studies [21,22,23,24,25,26,27,28,29,30] that explored the effects of hypoglycemic medications on cardiac reverse remodeling, specifically measuring changes in LVESD and LVEDD. The research designs included randomized, double-blind, placebo trials [21,26,29], randomized prospective open-label blinded-endpoint trials [22,28,30], and prospective observational studies [23,24,25,27]. Study quality assessments classified several as high and others as medium. Notably, the studies by Chen et al. [21], Yamada et al. [22], Yamamoto et al. [23], Sardu et al. [24], Otagaki et al. [25], Rau et al. [26], Gamaza-Chulian et al. [27], Reis et al. [28], Fu et al. [29], and Hong et al. [30] represented a significant cross-section of international research, indicating broad global interest and diverse applications of hypoglycemic medications in cardiac care across different patient populations.

### 3.2. Results of Individual Studies

Across the ten studies included in the systematic review, a total of 1180 patients were analyzed. The average age of these patients was 69.1 years, while the proportion of male participants was about 70.7%. These studies varied considerably in sample size, ranging from 40 [28] to 559 participants [24], predominantly involving elderly subjects with average ages spanning from 57.7 [21] to 72 years [24]. The medications reviewed included Glucagon-like Peptide-1 Receptor Agonists (GLP-1RA), Dipeptidyl Peptidase-4 Inhibitors (DPP-4I), and Sodium Glucose Cotransporter Type 2 Inhibitors (SGLT-2I), with the patient populations mostly composed of men and the gender proportion varying from 55.8% [27] to 82.5% [28]. Comorbidities across these cohorts included cardiovascular disease (CVD), either alone or combined with Type 2 Diabetes Mellitus (T2DM), highlighting the prevalent cardiovascular risk factors in the studied groups, as presented in Table 2.

### 3.3. Results of Synthesis

In a study by Chen et al. [21], Liraglutide showed a notable improvement in LVEF from 47.2% to 57.2% after three months, which was statistically significant compared to the placebo group. Similarly, both LVEDD and LVESD showed reductions, indicating a positive effect on cardiac dimensions and function in patients with Non-ST-Elevation Myocardial Infarction (NSTEMI). Conversely, Yamada et al. [22] reported minimal changes in cardiac measurements after 24 months of Sitagliptin treatment, with no significant impact on cardiac reverse remodeling, except for some improvement in diastolic function markers.

Other studies have demonstrated varied responses depending on the treatment duration and drug type. For example, Sardu et al. [24] found that treatment with GLP-1 RA over 12 months resulted in improvements in all measured cardiac dimensions, alongside a decrease in arrhythmic events and heart failure hospitalizations, highlighting the potential of this treatment in enhancing cardiac outcomes. On the other hand, treatments with Dapagliflozin in studies by Fu et al. [29] and Hong et al. [30] over 12 months not only showed significant improvements in all cardiac dimensions but also demonstrated the efficacy of long-term treatment in promoting beneficial cardiac remodeling, particularly in patients with Heart Failure with reduced ejection fraction (HFrEF) and non-ischemic dilated cardiomyopathy (NIDCM).

**Figure 2:**
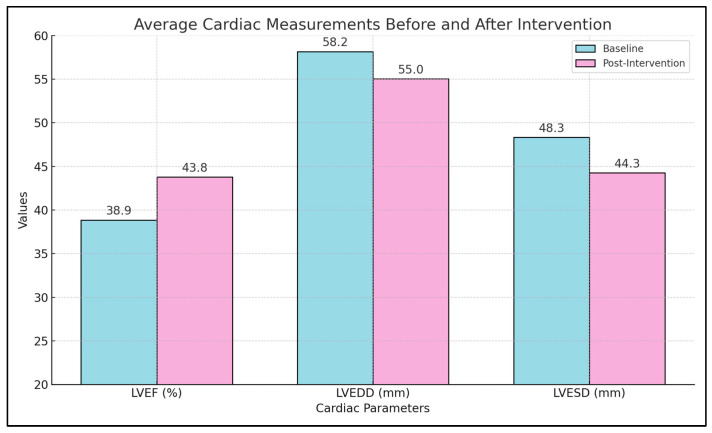
Average cardiac measurements before and after intervention.

The average cardiac measurements showed a significant improvement in LVEF after intervention, from a mean value of 38.9% to 43.8%. Similar significant findings were identified in terms of LVEDD (from a baseline of 58.2 mm to 55.0 mm after intervention), and LVESD (from a baseline of 48.3 mm to 44.3 mm after intervention). These results collectively suggest that while some treatments like Sitagliptin may offer limited cardiac remodeling benefits, others like Liraglutide, Dapagliflozin, and GLP-1 RA appear to significantly enhance both the structural and functional aspects of the heart (Table 3 and Figure 2).

## 4. Discussion

### 4.1. Summary of Evidence

This systematic review critically evaluated the impact of hypoglycemic medications on cardiac reverse remodeling, particularly focusing on left ventricular dimensions in patients with diabetes mellitus. Among the notable findings, Liraglutide demonstrated a profound improvement in left ventricular ejection fraction, from 47.2% to 57.2%, and reductions in LVEDD and LVESD within three months of treatment. These results are consistent with prior studies suggesting that GLP-1 receptor agonists, like Liraglutide, may exert significant cardioprotective effects by improving cardiac function and structure, potentially via mechanisms linked to improved metabolic control and direct cardiovascular actions.

Conversely, the minimal changes observed with Sitagliptin over 24 months highlight the variability in the cardiac effects of DPP-4 inhibitors, which have been suggested to have more modest benefits on cardiac remodeling. This discrepancy may stem from differences in the molecular mechanisms of action between GLP-1 receptor agonists and DPP-4 inhibitors, where the former has a more direct role in cardiovascular protection. The differential impact underscores the importance of selecting appropriate hypoglycemic treatments based on individual patient cardiovascular risk profiles and the specific cardiac benefits of each drug class.

The significant improvements observed with Dapagliflozin in both short-term and long-term studies, particularly in patients with heart failure with reduced ejection fraction (HFrEF) and non-ischemic dilated cardiomyopathy (NIDCM), further support the role of SGLT-2 inhibitors in cardiac reverse remodeling. These findings align with the growing body of evidence that SGLT-2 inhibitors not only improve glycemic control but also confer substantial cardiovascular benefits, possibly through effects on cardiac load, metabolic efficiency, and direct myocardial energetics.

The systematic reviews by Huang et al. [17] and Carluccio et al. [18] provide comparative insights into the effects of hypoglycemic agents and SGLT2 inhibitors on cardiac remodeling, although focusing on different cardiac measurements than in our systematic review where the LVEDD and LVESD were the main study outcomes. Huang et al. found that GLP-1RA notably improved left ventricular end-systolic diameter by −0.38 mm and mass index (LVMI) by −1.07 g/m^2^, contrasting with DPP-4i, which enhanced diastolic function but reduced left ventricular ejection fraction by −0.89%. SGLT-2i, on the other hand, improved both LVMI by −0.28 g/m^2^ and left ventricular end-diastolic volume by −0.72 mL, indicating broad benefits. Carluccio et al. highlighted the efficacy of SGLT2 inhibitors in heart failure patients, showing significant reductions in LVEDV by −10.59 mL, LVESV by −8.80 mL, and an increase in LVEF by +1.98%.

Animal studies such as those by Ting-I Lee et al. [31] and Camila Moreno Rosa et al. [32] explored the cardioprotective effects of SGLT2 inhibitors using streptozotocin-induced diabetic rat models, highlighting similar yet distinct outcomes. Lee et al. reported that empagliflozin treatment led to decreased left ventricular end-diastolic diameters and shorter QT intervals, with significant improvements in the electrophysiological properties of cardiac cells—such as reduced late Na+ current and Na+/hydrogen-exchanger currents. This study detailed cellular-level enhancements, like the reduction in the incidence and frequency of Ca2+ sparks and a decrease in oxidative stress markers. On the other hand, Rosa et al. found that Dapagliflozin not only improved cardiac dimensions but also led to notable systemic effects, including a decrease in blood pressure and glycemia, and an increase in body weight (from 381 ± 52 g in diabetic rats to 430 ± 48 g in treated rats). Their study emphasized broader cardiovascular improvements, noting an attenuation in myocardial hydroxyproline concentration and a lower lipid hydroperoxide concentration (304 ± 40 nmol/g tissue in treated rats versus 385 ± 54 nmol/g in untreated). While Lee et al. focused more on the stabilization of cardiac function through modulation of ionic currents, Rosa et al. highlighted the reduction of oxidative stress and an overall improvement in cardiac structural function, making both studies complementary in illustrating the multi-faceted benefits of SGLT2 inhibitors in diabetic cardiomyopathy.

Similarly, Ikonomidis et al. [33] demonstrated significant improvements in vascular and myocardial function markers after a 12-month treatment with GLP-1RA and SGLT-2 inhibitors, or their combination. They reported notable reductions in the perfused boundary region (improved by over 10% with combination therapy), pulse wave velocity, and central systolic blood pressure, with the myocardial work index increasing by 12.7% and 17.4% in GLP-1RA and the combined therapy groups, respectively. Conversely, Paiman et al. [34], focusing on South Asian patients in the Netherlands treated with Liraglutide, observed no significant changes in left ventricular diastolic and systolic function, with stroke volume decreasing by 9 mL and heart rate increasing by 10 bpm. These disparities highlight the influence of patient demographic and treatment modality on therapeutic outcomes, where the combination therapy in a diverse European cohort showed superior efficacy compared to monotherapy in a specific ethnic group.

The studies by Santos-Gallego et al. [35] and Akasaka et al. [36] explored the impact of SGLT2 inhibitors on cardiac function, albeit with different patient profiles and endpoints. In the EMPA-TROPISM study by Santos-Gallego et al., empagliflozin significantly improved cardiac structure and function in nondiabetic patients with heart failure and reduced ejection fraction (HFrEF), evidenced by marked reductions in left ventricular end-diastolic volume (−25.1 mL) and end-systolic volume (−26.6 mL), enhancements in ejection fraction (+6.0%), and improvements in functional capacity measures such as the 6 min walk test (+81 m) and peak oxygen consumption (+1.1 mL/min/kg). Contrastingly, Akasaka et al., in the EXCEED study, did not observe significant improvements in diastolic function in diabetic patients with heart failure and preserved ejection fraction (HFpEF) after treatment with ipragliflozin, with minimal changes in E/e’ and other diastolic function parameters over 24 weeks. These contrasting outcomes underline the potential of SGLT2 inhibitors like empagliflozin in improving systolic and volumetric parameters in HFrEF, which may not translate similarly to HFpEF patients, particularly those managed with diastolic function as a primary outcome.

### 4.2. Limitations

This study posits that reverse remodeling improves outcomes; however, this remains a hypothesis in the context of diabetic cardiomyopathy. The lack of definitive evidence supporting this relationship necessitates a cautious interpretation of the findings and the acknowledgment of this as a limitation. Several other limitations that could affect the interpretation and generalizability of the findings are necessary to acknowledge. Primarily, the variability in study designs, patient populations, and treatment durations across the included studies introduced a degree of heterogeneity that may limit the applicability of the results. Additionally, the predominance of older adult participants with comorbid cardiovascular conditions in most studies may not have fully represented outcomes in younger populations or individuals without significant cardiac histories. Furthermore, the mixed quality of the studies, with some classified as medium, indicates potential biases that could impact the robustness and reliability of the synthesized data.

## 5. Conclusions

In conclusion, this systematic review demonstrates that certain hypoglycemic medications, particularly Liraglutide and Dapagliflozin, can significantly improve cardiac dimensions and functions in patients with diabetes, suggesting their potential utility in managing cardiac comorbidities associated with diabetes. The evidence supports a nuanced approach to prescribing these medications, tailored to the individual patient’s cardiovascular risk and existing comorbidities, to maximize both glycemic and cardiac outcomes. Further high-quality, long-term randomized controlled trials are needed to expand upon these findings and better define the mechanisms through which these medications exert their cardioprotective effects.

## Figures and Tables

**Table 1:** Study characteristics.

Number	First Author	Reference	Country	Study Year	Study Design	Study Quality
1	Chen et al.	[21]	China	2015	Randomized, double-blind, placebo trial	High
2	Yamada et al.	[22]	Japan	2017	Randomized, prospective, open-label, blinded-endpoint trial	High
3	Yamamoto et al.	[23]	Japan	2017	Prospective, observational	Medium
4	Sardu et al.	[24]	Italy	2018	Prospective, observational	Medium
5	Otagaki et al.	[25]	Japan	2019	Prospective, observational	Medium
6	Rau et al.	[26]	Germany	2021	Randomized, double-blind, placebo trial	High
7	Gamaza-Chulian et al.	[27]	Spain	2021	Prospective, observational	Medium
8	Reis et al.	[28]	Portugal	2022	Randomized, open-label trial	Medium
9	Fu et al.	[29]	China	2023	Randomized, double-blind, placebo trial	High
10	Hong et al.	[30]	China	2024	Randomized, prospective, open-label, blinded-endpoint trial	Medium

**Table 2:** Characteristics of patients included in the studies.

Number	First Author	Reference	Sample Size	Age (Years)	Treatment	Comorbidities	Gender (Men)
1	Chen et al.	[21]	83	57.7 (11.3)	GLP-1RA	CVD	73.3%
2	Yamada et al.	[22]	115	69 (8)	DPP-4I	T2DM	67.0%
3	Yamamoto et al.	[23]	137	71 (10)	DPP-4I	CVD+T2DM	63.8%
4	Sardu et al.	[24]	559	72 (6)	GLP-1RA	CVD+T2DM	72.1%
5	Otagaki et al.	[25]	42	65 (13.8)	SGLT-2I	T2DM	69.1%
6	Rau et al.	[26]	42	62 (6.8)	SGLT-2I	T2DM	77.2%
7	Gamaza-Chulian et al.	[27]	52	66.8 (8.6)	SGLT-2I	T2DM	55.8%
8	Reis et al.	[28]	40	60.9 (13)	SGLT-2I	CVD	82.5%
9	Fu et al.	[29]	60	70.7 (6.7)	SGLT-2I	CVD+T2DM	71.7%
10	Hong et al.	[30]	50	67 (59.7)	SGLT-2I	CVD	80.0%

NR—not reported; T2DM—Type 2 Diabetes Mellitus; CVD—cardiovascular disease; GLP-1RA—Glucagon-like Peptide-1 Receptor; DPP-4I—Dipeptidyl Peptidase-4 Inhibitor; SGLT-2I—Sodium Glucose Cotransporter Type 2 Inhibitor.

**Table 3:** Cardiac measurements and outcomes.

Number	First Author	Reference	Treatment Scheme	LVEF	LVEDD	LVESD	Outcomes and Interpretation
1	Chen et al.	[21]	Liraglutide for 3 months	Placebo: 47.7% at baseline vs. 52.6% at 3 months. Liraglutide: 47.2% at baseline vs. 57.2% at 3 months *.	Placebo: 46.3 mm at baseline vs. 45.9 mm at 3 months. Liraglutide: 46.5 mm at baseline vs. 45.2 mm at 3 months.	Placebo: 35.1 mm at baseline vs. 33.7 mm at 3 months. Liraglutide: 35.2 mm at baseline vs. 32.7 mm at 3 months *.	Liraglutide could improve left ventricular function in patients with NSTEMI.
2	Yamada et al.	[22]	Sitagliptin for 24 months	Baseline: 63.6%; 24 months: 62.5%	Baseline: 48.5 mm; 24 months: 47.5 mm	Baseline: 31.7 mm; 24 months: 31.5 mm	Except for significant e baseline-adjusted change in E/e′ for 24 months, Sitagliptin treatment was not associated with improvements in cardiac reverse remodeling.
3	Yamamoto et al.	[23]	DPP-4I (not mentioned) for 12 months	Baseline: 45%; 12 months: 50% *	Baseline: 55.7 mm; 12 months: 53.8 mm *	NR	DPP-4i treatment over 1 year showed improvement in LVEF and a decrease in LVEDD, suggesting potential stabilization or improvement in cardiac structure and function in HFpEF patients.
4	Sardu et al.	[24]	GLP-1 RA (not mentioned) for 12 months	Baseline: 27%; 12 months: 32% *	Baseline: 67 mm; 12 months: 66 mm	Baseline: 43 mm; 12 months: 36 mm	CRTd with GLP-1 RA therapy led to improvements in LVEF, reduced LVEDD and LVESD, significant decreases in arrhythmic events and heart failure hospitalizations, and an increase in CRTd responders.
5	Otagaki et al.	[25]	Tofogliflozin for 6 months	Placebo: 61.0% at baseline vs. 63.0% at 6 months. Tofogliflozin: 58.0% at baseline vs. 63.0% at 6 months *.	Placebo: 48.0 mm at baseline vs. 49.0 mm at 6 months. Tofogliflozin: 50 mm at baseline vs. 45 mm at 6 months *.	Placebo: 34 mm at baseline vs. 32 mm at 6 months. Tofogliflozin: 32 mm at baseline vs. 30 mm at 6 months.	Tofogliflozin treatment over 6 months demonstrated significant improvements in LVEF, LVEDD, and E/e’, suggesting beneficial effects on both systolic and diastolic functions in T2DM patients.
6	Rau et al.	[26]	Empagliflozin for 3 months	Placebo: 48% at baseline vs. 48% at 3 months. Empagliflozin: 51% at baseline vs. 51% at 3 months.	Placebo: 50 mm at baseline vs. 50 mm at 3 months. Empagliflozin: 49 mm at baseline vs. 48 mm at 3 months.	Placebo: 36 mm at baseline vs. 37 mm at 3 months. Empagliflozin: 34 mm at baseline vs. 33 mm at 3 months.	No significant changes in LVEF, LVEDD, or LVESD were observed over the course of treatment with empagliflozin compared to placebo.
7	Gamaza-Chulian et al.	[27]	SGLT-2I (not mentioned) for 6 months	Baseline: 64.6%; 6 months: 64.4%	Baseline: 44.5 mm; 6 months: 45.0 mm	NR	SGLT2 inhibitors were associated with significant reductions in indexed LVM and improvements in GLS, suggesting beneficial effects on cardiac remodeling and function in T2DM patients, although there was no significant evidence of reverse remodeling in terms of LVEF and LVEDD.
8	Reis et al.	[28]	Dapagliflozin for 6 months	Baseline: 34.5% vs. 6 months: 36.9% (Dapagliflozin) Baseline: 33.5% vs. 6 months: 36.2% (Control)	Baseline: 65.1 mm vs. 6 months: 63.0 mm (Dapagliflozin) Baseline: 67.2 mm vs. 6 months: 65.8 mm (Control)	Baseline: 49.4 mm vs. 6 months: 46.1 mm (Dapagliflozin) Baseline: 46.9 mm vs. 6 months: 41.6 mm (Control)	There were no statistically significant changes in LVEF, LVEDD, or LVESD observed over the course of treatment with Dapagliflozin compared to the control. The study suggested a modest trend towards improved cardiac function and remodeling in the Dapagliflozin group, although changes were not statistically significant.
9	Fu et al.	[29]	Dapagliflozin for 12 months	Baseline: 30.6% vs. 12 months: 36.3% (Dapagliflozin) Baseline: 31.3% vs. 12 months: 33.7% (Placebo)*	Baseline: 59.3 mm vs. 12 months: 53.8 mm (Dapagliflozin) Baseline: 59.9 mm vs. 12 months: 55.5 mm (Placebo) *	Baseline: 50.3 mm vs. 12 months: 46.0 mm (Dapagliflozin) Baseline: 50.7 mm vs. 12 months: 48.3 mm (Placebo) *	Dapagliflozin demonstrated a statistically significant improvement in LVEF, LVEDD, and LVESD over one year, suggesting beneficial effects on cardiac remodeling in patients with type 2 diabetes and HFrEF.
10	Hong et al.	[30]	Dapagliflozin for 12 months	Baseline: 27.87% vs. 12 months: 42.22% (Dapagliflozin) Baseline: 31.99% vs. 12 months: 35.52% (Placebo)*	Baseline: 64.3 mm vs. 12 months: 59.35 mm (Dapagliflozin) Baseline: 68.30 mm vs. 12 months: 66.37 mm (Placebo) *	Baseline: 53.61 mm vs. 12 months: 47.39 mm (Dapagliflozin) Baseline: 57.15 mm vs. 12 months: 54.52 mm (Placebo) *	Dapagliflozin combined with conventional therapy resulted in significant improvements in LVEF, LVEDD, and LVESD compared to placebo, indicating a substantial benefit in cardiac function and remodeling in NIDCM patients.

NR—not reported; LVEF—left ventricle ejection fraction; LVEDD—left ventricle end-diastolic diameter (mm); LVESD—left ventricle end-systolic diameter (mm); *—statistically significant differences; GLP-1RA—Glucagon-like Peptide-1 Receptor; DPP-4I—Dipeptidyl Peptidase-4 Inhibitor; SGLT-2I—Sodium Glucose Cotransporter Type 2 Inhibitor; LVM—Left Ventricular Mass; GLS—Global Longitudinal Strain.

## Data Availability

The original contributions presented in the study are included in the article; further inquiries can be directed to the corresponding author.
